# Study on the effects of sustainability practices on the growth of manufacturing companies in urban Ghana

**DOI:** 10.1016/j.heliyon.2019.e01903

**Published:** 2019-06-21

**Authors:** Kwame B. Bour, Akowuah Jones Asafo, Bernard Owusu Kwarteng

**Affiliations:** a Department of Sociology and Social Work, Spiritan University College, Kumasi, Ghana; b Department of Agricultural Economics, Agribusiness and Extension, Kwame Nkrumah University of Science and Technology, Kumasi, Ghana; c Department of History and Political Studies, Kwame Nkrumah University of Science and Technology, Kumasi, Ghana

**Keywords:** Sustainability practices, Corporate sustainability performance, Manufacturing companies, Ghana, Environmental science

## Abstract

Manufacturing companies have come under intense pressure to sustain the environment in which they operate. This has led to the adoption of ‘greener’ and more environmentally friendly approaches among firms. This paper explores the effects of sustainability practices on the growth of manufacturing companies in urban Ghana involving six manufacturing companies. Using the cross-sectional design with a two-stage sampling technique, 600 respondents were selected through structured questionnaires across 6 companies. The empirical model was tested using regression analysis, to verify the hypothetical relationships of the study. The results of this study indicate model Summary with R Square of 0.813 shows that environmental sustainability practices of manufacturing companies have significant positive profit margins in their operational activities. Again, regression coefficients of the predictor variables are at 1% and 5% significance. The (2-tailed) Pearson Correlation between cost of Maintaining ESPs and the Plans for Improving ESPs is significant at 1%. Further sustainability practices are suggested to strengthen the operations of manufacturing companies.

## 1 Introduction

In recent times, environmental problems have become so profound that disciplines such as Sociology, which hitherto, did not consider physical environment as part of their subject matter, now considers it obligatory to do so (Dunlap and Catton, 1979 and Brym, 2001). These environmental concerns have been thoroughly discussed and deliberated on in most international fora primarily due to globalization (Cramer, 2017; Tukker et al., 2018). Probably the dialectical relationship between physical environment and human activities has now become more visible. Moreover, the 1970s saw an increase in urban decay, pollution, overpopulation, resources shortage, ozone layer depletion and general environmental degradation (Buttel, 2003).

There is therefore the global call on companies to be registered under ISO 14001 and implement all accompanied guidelines to ensure environmentally friendly practices (Nawrocka and Parker, 2009). These regulatory and innovative practices not only help to ensure internal sanity among companies but also throughout the supply chains of their entire operations (Bansal, 2005; Hansen et al., 2009). Hence, these management practices assume the broader stakeholder concept on corporate sustainability (Hörisch et al., 2014). According to Barr (2016), the period marked a turning point in human conceptualisation of the environment. Since it became a cause for concern, many scholars from different fields of study redirected their interest and focus onto environmental discourse analysis in an attempt to mitigate anthropogenic assault on the environment. The major concern of environmental ethics since then has been to develop environmental theory of value (Freeman et al., 2014). As Goodin (1992), cited in Carter (2001) describes it as a theory of good which tells what is to be valued and why in respect of the environment.

Environmental problems have also been severe in Africa and that Ivory Coast and Ghana's Cocoa production is even under threat of collapse by 2050 (Beinart, 2000). Kouakou (2013) also lends support to this claim of industrial assault on the environmental sustainability in Africa by indicating that indiscriminate industrial activities threaten environment in Ivory Coast. The state of the environment in Ghana has also not been the best even though UNEP (2013) declared that Ghana has a relatively clean atmosphere. However, the gloomy picture is that between 2008 and 2011, the manufacturing sector contributed about 1.7% to GDP but the environmental cost of this sector is estimated at 10% of the GDP (CPA, 2011; UNEP, 2013). Currently, according to the same UNEP report industrial sector including manufacturing subsector is the leading contributor of all GHG emissions in Ghana.

Since then, Ghana has ratified almost all the UN conventions relating to the environment, including the United Nations Framework Convention on Climate Change (UNFCCC), according to which national government is expected to take climate change issues into consideration in its development planning. To demonstrate the commitment of the Government of Ghana to environmental protection and sustainability, Act 490 established Environmental Protection Agency (EPA), formerly called Environmental Protection Council, in 1994 to regulate all economic or otherwise activities that affect the environment in anyway. A subsequent Law; Environmental Assessment and Regulations (LI.1652) was passed. These legal instruments made it mandatory for companies and enterprises to submit their environmental management plans or intended practices as a requirement for registration. The already existing ones are also expected to conduct annual environmental impact assessment for consideration by the EPA.

Environmental problems is said to be widespread in manufacturing areas all over the world and that there is a growing concern about the ever-increasing environmental degradation due to industrial activities (Kpelle et al., 2014; Ramos et al., 2018; Sarkis, 2017; Masrom et al., 2018). Many manufacturing industries discharge organic and inorganic wastes into the environment especially into water bodies. These include acids, highly toxic minerals, such as mercury or arsenic, or toxic organic chemicals. Such environmental injustice can have negative effects such as rendering water unsafe for domestic use and even for irrigation. The pollutants may enter the food chain and cause health complications for humans (Carter, 2001).

Karcagi-Kovats (2012) has stated that the most serious and urgent task of humanity is to avert the global ecological crisis and several other dangers which lurk on them such as mass famine and the overturn of the dynamic balance of the planet. If nothing is done, the environment is most likely to experience devastation together with the populations thereon. The severity of the effects of the manufacturing industry on the environment is expressed in GEO 4 report that, human activities especially industrial activities including manufacturing are responsible for the production of GHGs, which increased the global temperature by 0.74%. This global warming gives rise to sea-level rise, increasing frequency of heat, storms, flood and drought. The report estimates that global warming is likely to be between 1.8 and 4^°^c by 2050 if the current attitude toward the environment remains constant.

Furthermore, Sarkis (2017); Forsyth (2002) reiterate that there is a lack of attention to brown environmental issues and that much attention has concentrated on green environmental agenda. Brown environmental agenda according to Sarkis and Forsyth focuses on environmental issues associated with urban and industrial locations such as pollution, waste disposal, and bad farming practices among others. Therefore, there is an urgent need to redirect attention of researchers to brown environmental agenda.

Ghana has committed herself to the achievement of the Millennium Development Goal 7 that is to attain sustainable environment. However, the environmental performance of Ghana in terms of efforts toward achieving environmental sustainability does not look too good even though there has been a reduction in GHG emissions (UNEP, 2000). For instance, the 2010 Environmental Performance Index of Yale University that ranked 163 countries on 25-performance indicator scale scored Ghana 51.3% and ranked her 109^th^. The report further indicates that the mean estimated annual cost of environmental degradation is nearly US $850 million or 10.0% of GDP. UNEP (2013) report also states that industrial sector including manufacturing sub-sector is the leading contributor of all GHG emissions in Ghana. Songsore (2004) had earlier indicated that industrial production brings with it the need to control and manage wastes from production sites for the protection of local ecology and health.

Following these ecological experiences are a series of ‘how’ questions which need critical examination and determination. The baseline level for establishing manufacturing companies in Ghana is that, companies submit their environmental sustainability practices and plans as mandatory requirement for the registration of such companies. Equally important is the fact that previous studies such as those conducted by Amanor and Brown (2003); Marfo and Anchirinah (2004); the World Bank (2006); Akuoko and Bour (2013); Kpelle et al. (2014) with which none focused on how manufacturing companies have not kept faith with environmental sustainability practices. In their work on making environmental management more responsive to local needs, Amanor and Brown (2003) restricted their work on decentralised policies of Ghana. Marfo and Anchirinah (2004) limited their study to forest areas with Akuoko and Bour (2013) focusing their studies on mining areas. Again, the work of Kpelle et al. (2014) was limited due to geographical location.

So by this requirement one expects every manufacturing company to be on top of environmental sustainability practices; now how come that these same companies fail the test on the implementation of the very thing they professed to apply at the workplace? Is it because, this is a mere requirement to present such documents for incorporation? How have companies failed the test they have set for themselves? Does that mean that what they present to the EPA is different from what they actually practice? Something is missing; that social fact, that explanation is needed urgently by means of a scientific research. There is therefore a huge gap between the act of obtaining and signing on to certification on environmental management practices and strictly adhering to the guidelines of such packages. Hence, this research is necessitated by this missing link to fill the gap. It is therefore, the attempt to respond to these ‘how’ questions and to fill that gap in the literature on environmental sustainability practices that necessitated this study.

## 2 Materials

### 2.1 Study setting

Ashanti region, which is the target region of this study, is centrally located in the middle belt of Ghana and lies between longitudes 0.15W and 2.25W, and latitudes 5.50N and 7.46N. It shares boundaries with four of the ten political regions including Brong-Ahafo to the north, Eastern region to the east, Central region on the south and Western region in the Southwest. The region is said to occupy a total land area of 24,389 square kilometres representing 10.2 per cent of the total land area of Ghana. It is also the most populous region with a population of 4,780,280, representing 19.4 percent of the country's total population (GSS, 2012) and a population density of 148.1 persons per square kilometres. More than half of the region's territory lies within the wet, semi-equatorial forest zone. History has it that the region was carved out of the Asanteman (Ashanti nation) founded during the reign of King Osei Tutu I (1697–1718) with Kumasi as its administrative capital (GSS, 2012). As of 2016, the region had 30 districts including 4 municipalities and Kumasi metropolis with 47 constituencies. The traditional head of the region is the Asantehene but the political head is the Regional Minister.

The target industrial sector of this study was the manufacturing sector in the Ashanti Region of Ghana. Manufacturing companies in the Ashanti Region were chosen for this study due to their abysmal performance in environmental sustainability practices assessment according to the EPA (EPA-AKOBEN, 2012). There were other economic sectors in the region whose activities also affected the physical environment badly and needed to have been studied though, the EPA (2012) report indicated that manufacturing organisations in the Region grossly failed to meet environmental sustainability standards. Ironically, these organisations profess to have environmental sustainability practices.

For the purpose of this study, only those companies rated red by the EPA in 2012 were selected except Coca-Cola Bottling Company, Ghana which was selected even though it was rated satisfactory. It was meant to serve as a control group because it had best practices. Moreover, since these organisations are concentrated in Kumasi, five of them were selected from within Kumasi and one was selected from Juabeng. They were selected based on the major manufacturing industries in the region that they belong to. The organisations were clustered into industries engaging in wood processing, chemical production, agro business, food, oil and drinks processing. The classification was done in accordance with the advice of the public relations officers of EPA in Kumasi. From these Clusters, the study organisations were then selected purposively from a list of 14 organisations listed with EPA-Ghana for environmental assessment in 2012. (see Tables 1 and 2)

**Table 1:** List of selected organisations.

Type of Manufacturing org.	Name of the organisation.	Staff strength
Wood Processing	Bibiani Logs and Lumber Co. Ltd (BLLC)	300
Animal Feeds Processing	Agricare Co. Ltd (AC)	186
Chemical Production	AmponsahEffa Pharmaceuticals Ltd (AEP)	224
Food Processing	Kumasi Abattoir (KA)	159
Oil Processing	Juabeng Oil Mills (JOM)	187
Drinks and Beverages	Coca-Cola Bottling Co. Gh (CBC)	360
Total		1416

Source: Authors' Construct

**Table 2:** Sample size for each of the selected companies.

Organisation	Ni	N|n	$\text{ni}\;=\;\frac{\text{Ni}}{\text{N}}\cdot \text{n}$
BLLC	300	1416|600	127
AC	186	1416|600	79
AEP	224	1416|600	95
KA	159	1416|600	67
CBC	360	1416|600	153
JOM	187	1416|600	79

Source: Authors' Construct

### 2.2 Study design and sampling

The study forms part of a larger research that assessed the effects of environmental sustainability practices on the growth of manufacturing companies in the Ashanti region of Ghana. The study adopted an organisation based survey design of six manufacturing companies owing to the ‘how’ and to ‘what’ extent of sustanaibility practices among manufacturing companies have had on the environment of their operations. Again, the study focuses on issues such as green technologies and environmental sustainability of manufacturing companies, which are all contemporary, hence, survey design was desirable (Creswell and Creswell, 2017). According to Nsowah-Nuamah (2005), surveys are among the systematic means of obtaining standardised information about the behaviour, attitudes and any other characteristics of a population being studied.

In order to obtain answers to the research questions raised and at the same time achieving the objectives of the study and drawing valid conclusions, the researchers took into account a sample that was representative. A representative sample according to Babbie (2005) is a sample that bears all the characteristics of the population it is representing.

According to Frey et al. (2000), survey designs make use of large populations which are reduced step by step to samples. Such steps involve the identification of unit of analysis, specification of target and study populations, sample size determination and sample selection. All these processes were embarked upon in order that sampling errors would be minimised.

Nevertheless, determining a sample size that would be equally representative of the population was also difficult. As a result, factors such as the population homogeneity or otherwise, fraction of the population constituting the sample and lastly the desired precision (margin of error) were considered in the determination of the sample size. Giving these factors, the margin of error for the determination of the sample size was 0.02 and the proportion of the study population likely to agree with the statement that environmental sustainability is a priority in the organisation was assumed to be 60% (0.60). The figures were then substituted into Moser and Kalton's (2017) formula for determining sample size. The formula is given as:

$$\frac{\text{n}={\text{P}}^{1}\;\left(1-{\text{P}}^{1}\right)}{{\left[{\text{SE}}_{\text{p}}\right]}^{2}}$$

where P^1^ = estimate of proportion of the population with a particular characteristic.

SEp = acceptable margin of error.

n = sample

$$\frac{\text{n}=0.60\;\left(1-0.60\right)}{{\left[0.02\right]}^{2}}$$

Therefore, the sample size for the study was 600.

The fractional approach is was used; it suggests that any fraction of the population that is 10% or more is deemed large enough to be a representative sample, especially if the sample is scientifically selected (Monette, 2005 cited in Babbie, 2005). Furthermore, Burns and Bush (2000) concluded that a sample size of 5% of a population of study is likely to achieve its purpose if it was scientifically chosen. The sample size of the study was about 42.4% of the total study population of 1,416. For this reason, the sample size of 600 respondents made up of the management, senior and junior staff of the selected organisations is considered adequate for the study. This margin of error decreases as the sample size increases beyond 384. Therefore, this sample size is considered adequate to yield the expected result, more especially, where the sample was selected scientifically by means of stratified sampling technique.

### 2.3 Sampling technique

In the first stage, all the targeted MCs were clustered under the major manufacturing industries in the region that they belong to. They were companies engaging in wood processing, pharmaceutical, animal feeds processing, food and meat processing, drinks and beverages as well as oil processing. Cluster sampling was used to select the five manufacturing companies in the Ashanti region which were listed by the EPA as the worse performing organisations in environmental sustainability practices. The remaining organisation (Coca-Cola Bottling Co.) was purposively chosen because it was the only manufacturing company whose operations in the region were rated satisfactory. Therefore, its inclusion was meant to serve as a control group to the other five organisations. The MCs were thus selected based on the major manufacturing industries in the region that they belong to. As such each company selected represented a cluster of companies in the manufacturing sector with common environmental sustainability practices in accordance with the EPA specifications. Furthermore, the choice of different companies was based on the assertion that using exclusively, organisations which have implemented environmental sustainability practices would mean that the sample will not vary on sustainability practices (Christmann, 2000).

In the second stage of the sampling process, proportional stratified sampling (Proportionate sample allocation) technique was used after clustering the manufacturing companies into wood processing, pharmaceutical, animal feeds processing, food and meat processing, drinks and beverages as well as oil processing. Akuoko (2008) has stated that when the population consists of a number of subgroups (strata) that are heterogeneous in nature, it is often desirable to use stratified sampling in order to cater for the variability in the population. It is a probability sampling design in which the population is first divided into homogenous strata and a sample is selected from each stratum. In stratified sampling researchers first divide the population into sub-groups and from each stratum they select a certain number of sample units to form the sample.

Bearing this in mind, a mathematical formula was applied in obtaining the proportion of each stratum (each company) of the study population to constitute the sample. The formula for determining the sample allocation for each stratum in the sample was given as:

$$\text{ni}\;=\;\frac{\text{Ni}}{\text{N}}\cdot \text{n}$$

i=1,2...6

$$\text{n}1\;=\;\frac{\text{N}1}{\text{N}}\cdot \text{n}$$

$$\text{n}2\;=\;\frac{\text{N}2}{\text{N}}\cdot \text{n}$$

$$\text{n}6\;=\;\frac{\text{N}6}{\text{N}}\cdot \text{n}$$

where Ni is the total population of each company.

N is the total population of the six (6) companies.

n is the sample size.

ni is the sample allocation for each stratum (company).

### 2.4 Data collection

Two instruments were used to capture information for analysis in the study. They were questionnaires and key-informants’ interviews. However, the main instrument used for the data collection was questionnaire. The questionnaire was the only quantitative data collection instrument used and was designed based on the objectives of the study. It comprised five sections; section A, B, C, D and E. of which section ‘A’ captured personal information of the respondents, section ‘B’ on sustainability practices and section C on challenges associated with maintaining standard environmental sustainability practices. Sections ‘D’ and ‘E’ captured data on the effects of environmental sustainability practices on the organisation and the management system for environmental sustainability. The questionnaire was made up of open and close ended questions. The close ended questions allowed respondents to answer question by making a choice out of a list of options. The open ended questions allows for flexibility in answering the question. It provides an opportunity for the respondents to provide their own responses to supplement the responses provided in the close ended questions. Finally Likert scaled type of questions were also used in order to help measure variables at the interval level.

Semi-structured interview schedules or protocols were designed to elicit information from the key-informants to support the data from the questionnaire survey. Notwithstanding, these two major data collection methods, direct observation was resorted to in certain situations to collect data to confirm the data gathered from the administration of the questionnaires and the key-informants’ interviews. This was in conformity to Bhattacherjee (2012 assertion that personal observation is important in social research.

### 2.5 Data collection and analysis

It is observed that the king pin of any scientific research is the accuracy of the analysis of its data. Burns and Bush (2000) agreed more when they said that it was the most important aspect of any research and ought to be done critically and technically. In this regard, data gathered were edited by scrutinising the completed questionnaires to make sure that the responses did not have errors so as to influence the validity of the results. Kothari (1994) indicated that editing of data involves critical examination of the raw data gathered from the field in order to identify errors and omissions and correct them to ensure that the data collected were accurate. Babbie (2005) believes that editing involves going through data in order to find out inconsistencies and errors. After editing, the responses to the open-ended questions were then coded just as the responses to the close-ended questions had been. The coding process involved categorising responses and assigning numbers to responses so that they can be put into one exclusive category. Coding was followed by the data cleaning. Cleaning data involved a critical search for coding errors identified as being impossible and improbably based on the way the variables were defined. Hereafter, a carefully scrutinised coding scheme was developed. The Statistical Package for Social Sciences (SPSS) software was used in processing quantitative data for analysis. As a result the variables and their coded attributes or values were keyed in the computer for SPSS software programme to process them for analysis. Both descriptive and inferential statistical components of SPSS were utilised. Through the descriptive statistics were presented using tables and chi-square with ANOVA were used to test for correlations and levels of significance.

### 2.6 Ethical approval

Since the study did not consider human experiment, no ethical approval was sought.

## 3 Results and discussion

### 3.1 Effects of ESPs on the growth of MCs

One of the main objectives of this study was to find out both positive and negative effects of ESPs on the growth of MFs in Ashanti region. The inclusion of this objective was meant to clarify uncertainty as to the real effects of ESPs on the growth of MCs. The conviction of the author of this study was that by ascertaining the real effects of ESPs would facilitate the conclusion as to why most of the MFs in Ashanti region failed to keep up with the environmental sustainability standards.

Elite theory of public policy according to Wilson (2006) assumes that business leaders and corporate interest dominate policy processes. Therefore, if these corporate bodies are fully aware of the effects of ESPs on the growth of their businesses they may or may not commit themselves to keeping best ESPs. They may employ all means to influence the direction of any policy that affects their interest. Some contemporary studies have concluded that ESPs have no effect on the growth of organisations in general. Organisations therefore, engage in ESPs as a matter of morality (Hibbert and Cunliffe, 2015). It is even believed that employees comply with ESPs for the sake of organisational citizenship (Barr, 2016). Others are also of the view that ESPs of organisations enhance their public relations leading to reduction in the cost of production (Landrum and Edwards, 2009). Swarbrooke Horner (2007) indicated that it creates competitive advantage for new market opportunities. Maas et al. (2016) on the other hand asserted that it leads to decrease in profit margin because of the cost involved.

This grey area needs a clarification in order to ascertain the real effects of ESPs on MFs in the Ashanti region, which will eventually facilitate the conclusion as to ‘how’ MCs have not committed to maintaining best ESPs. As a result, respondents were asked to indicate their level of agreement with some statements meant to test the effects of ESPs on the growth of MFs involved in the study. The Likert scale type of questions was designed to enable the variable ‘effects of ESPs on the growth of the MFs’ to be measured at the interval level. The variable was measured at that level so that means and standard deviations could be used to analyse the data. It is observed therefore, from Table 3 that ESPs have some effects on the growth of the MCs in the Ashanti region.

**Table 3:** Item Statistics on the effects of ESPs on the Growth of MFs.

	Mean	Std. Deviation	N
Environmental sustainability practices of the organisation affect the reputation people attach to the organisation	2.8221	1.20583	596
Environmental sustainability practices enhance business growth and development	2.8322	1.01520	596
Environmental sustainability practices ensure reduction of cost in the long run but increase cost in the short run	3.3758	.78410	596
Maintaining best environmental sustainability practices reduces the profit margin of the organisation	4.1930	.49333	596
Good environmental sustainability practices lead to positive public relations	3.2450	.49575	596
Good ESPs improve corporate image with stakeholders and the local community	3.6292	1.14279	596
Poor environmental sustainability practices result in conflict between the organisation and the community	3.2836	1.13986	596

Grand Mean = 2.9077.

Source: Authors' Construct

The grand mean (3.4830) was used as the base mean with which the means of the various effects were compared. By so doing, the magnitude of these effects was revealed. Hence it is seen in the table that maintaining best ESPs reduces the profit margin of the MFs (M = 4.1930; S = .4933). As a result, maintaining best ESPs has the greatest magnitude of the effects on the growth of the MFs. This implies that MCs which endeavour to keep best ESPs risk a decline in profit margin at least in the short run. This situation was confirmed by the northern sector manager of Coca Cola Ghana Limited in an exclusive interview. He said, "having best Environmental Sustainability practices is expensive".

In determining the level of correlation relating to the effect of the various ESPs on the profit margins of MCs involved in the study, linear regression was used for the prediction (Table 4). The dependent variable; profit margin of the firm was measured in percentages given by the respondents which were later on converted mechanically into log for the purpose of linearisation. The independent variable (the various ESPs) on the other hand was measured by ranking them on a scale of 1–5 (5 being the highest score and 1 being the least) because that variable had five attributes or indicators. It should be noted, however, that authors were mindful of the likelihood of respondents’ guessing of the effect of the ESPs on the profits of the companies, so they were asked to indicate the source of their information. To further validate the information, the finance officers of the six MCs involved in the study were called upon to authenticate the responses and where necessary some adjustments were made.

**Table 4:** Model summary of the effects of ESPs on the profit margin of MCs.

R	R Square	Adjusted R Square	Std error of the estimate
0.902	0.813	0.657	0.371

Source: Author's construct.

Table 4 presents a summary of the regression model employed in the analysis of the relationship between profit margin of the organisations and the commitment to the utilisation of the various ESPs. The R square of 0.813 means that the model explains about 81.3% of the variations in the dependent variable. In other words about 81.3% of the dependent variable is due to the independent variable (ESPs). Even where adjustments were made for errors, about 65.7 % of the variations in the dependent variable is accounted for by the independent variable. The study is, however, in consistent with the work of Sezen and Çankaya (2013) and Nallusamy et al. (2015) that green amanufacturing on corporate sustainability performance of manufacturing companies has direct effects on profit margins of companies. This study, therefore, agrees with the work of Zhu and Sarkis (2004), Rao and Holt (2005) that environmental sustainability practices (green initiatives) of manufacturing companies have significant positive profit margins in the operational activities of such companies, though Zhu et al. (2007) have found no positive correlation between green technologies of companies and their profit margins.

It is important to note that the negative coefficient implies that a 100% increase in the adoption of that particular practice will reduce the profit margin of the firm by a percentage point equivalent to the coefficient multiplied by100 (Table 5). However, the positive coefficient means that 100% increase in the use of that particular practice will increase their profit margin by a percentage point equivalent to the coefficient multiplied by 100.

**Table 5:** Regression coefficients of the predictor variable.

Predictor Variable	B	Std. Error	P-value
Constant	0.592	0.238	0.010
Traditional practices such as using chimneys, rubbish bin and clean-ups	0.041**	0.390	0.352
Installation of state of the art facilities to reduce pollution such as bio-digesters for treating of liquid waste, kilns for drying wood etc	-0.060***	0.020	0.003
The use of fume-free machinery such as LPG powered and energy conservation machines.	-0.103**	0.021	0.012
Environmental and waste management policy (reuse, recycling, waste reducing etc)	0.021**	0.003	0.000
Environmental socialisation (workshops, seminars, conferences etc) and Eco-awards.	-0.063**	0.025	0.021

Dependent variable: Log of profit margin (%), statistical significance: <0.01(***) and <0.05 (**).

Source: Authors' construct.

It is therefore observed in Table 5 that the organisations which engage in traditional ESPs such as using chimneys, rubbish bins and clean-ups are likely to increase their profit margin by 4.1%. However, the coefficient of 0.041 is not significant at 0.05 because the p-value was 0.352 which is greater than 0.05. This means that variability among scores is wide and the result may not be valid and reliable. This revelation is in agreement with the work of Vachon and Klassen (2006) and Nallusamy et al. (2015) which found no significant relationships between green technologies vis-a-vis performance outcomes and profit margins. Nevertheless, the practice has the tendency to increase the firm's profit margin perhaps due to low cost of engaging in such traditional practices and the public relations aspects of it. The observation confirms an earlier finding by Jasch and Savage (2005; Mayamurugan, 2016; Kaleel Ahmed et al., 2018) that environment-related cost is high and for that matter organisations engage in ESPs that are likely to increase or maintain their profit.

In addition, management practices among manufacturing companies were considered. Hence, the study sought to present a regression analysis on the installation of state of the art facilities to reduce pollution (such as bio-digesters for treating liquid waste, kilns for drying wood etc) and profit margin of firms. The result (Table 5) shows that companies, which install state of the art facilities to reduce pollution, are likely to decrease their profit margin by 6%. This relationship is inferred from the regression coefficient of -0.060, which is significant at 0.01 with a p-value of 0.003. Similarly, the Hannover Chamber of Commerce found that materials including environmental sustainability practices accounted for 40% of the costs in manufacturing industries as compared to labour, which accounted for 23% of the total cost (Jasch and Savage, 2005). Therefore, the prediction is more likely to be accurate. According to the United Nation's Division for Sustainable Development (2001), this evidence proves that the probability of losing 6% of the profit of the organisation to environmental sustainability is high and therefore, management is more likely to cut corners to avoid that cost. This could account for why MCs fail to meet EPA environmental sustainability standards because they would not like to risk losing about 6% of their profit.

On the case of the use of fume, free machinery such as LPG powered and energy conservation machines, the result from the regression analysis indicates that such a practice significantly reduces profit margin of companies. Statistically, the analysis reveals that 100% increase in the use of this practice reduces the profit margin of the organisations by 10.3% and this was significant at 0.05. The regression analysis also revealed that having environmental and waste management policy on reuse, recycling, waste reducing among other practices is likely to increase profit margin by 2.1%, which was significant at 0.05 with p-value of 0.000. Earlier studies have established that having environmental policy in an organisation would keep employees informed about their environmental roles and responsibilities, improving cost control, reducing incidents that result in liability, conserving raw materials and energy, improving monitoring of environmental impacts and lastly improving the efficiency of processes (Burden, 2010; Mayamurugan, 2016).

In view of the aforementioned benefits of having environmental policy, it is therefore, not out of place that this study discovered that having environmental and waste management policy increases profit by 2.1%. Furthermore, it is explained that environmental policies could stimulate innovation and investment in innovation by internalising the external costs of pollution and natural resource use. Policies could change relative prices and stimulate research and development as well as the uptake of alternative inputs, production methods and products.

Simultaneously, environmental policies lead to innovations in conservation of resources and energy; pollution prevention practices (PPPs) and environmental clean-up. These innovations were found to have the potential to reduce cost and reinforce the competitiveness of EU industries, because ‘clean’ technologies developed in Europe have become successful export products in the world (Ganesan et al., 2016; Basu et al., 2018). It has been established that policies induced by environmental innovations have directly and indirectly engendered, competitiveness, growth and job creation in the organisations (Rayment et al., 2009). These writers have observed that European Commission had estimated that the total commercial value of eco-innovative products and technologies in sustainable construction, renewable energy, bio-based products and recycling in the EU could grow from €92 billion in 2006 to €259 billion in 2020, thereby creating more than2.4 million new jobs.

Last but not least is the use of environmental socialisation (workshops, seminars, conferences etc) and Eco-awards strategies to achieve environmental sustainability. It also revealed that such practices are likely to decrease the profit of the organisation by 6.3%, significant at 0.05. This could be explained in terms of the cost involved in organising such events.

The analysis has so far established that some ESPs such as environmental and waste management policy on reuse, recycling, waste reducing etc and engaging in traditional ESPs such as using chimneys, rubbish bins and clean-ups are likely to increase profit margins. Hence, environmental socialisation (workshops, seminars, conferences) and eco-award strategies to achieve environmental sustainability, installation of state of the art facilities to reduce pollution and the use of fume-free machinery such as LPG powered and energy conservation machines on the other hand decrease profit margin of MFs. This observation therefore corroborates the previous findings by some environmental researchers including (Amanor and Brown, 2003; Akuoko and Bour, 2013; Barr, 2016; Sarkis, 2017) that ESPs have both positive and negative effect on the finances of firms and therefore, the growth of such organisations. Landrum and Edwards, 2009; Hitchcock and Willard and Hitchcock (2009) are even of the view that positive implications ESPs have on the growth of organisations are both short term and long term.

The next in magnitude in terms of the effects of the ESPs on the growth of MFs is the issue of good ESPs improving corporate image with stakeholders and local community (M = 3.6292; S = 1.1428) (Table 3). This advantage has always been the reason behind the call on firms to embark on best ESPs (Che, 2011; Kaleel Ahmed et al., 2018). Nevertheless, as to whether this advantage comes with financial realities is another matter as alluded to by the environmental officers of manufacturing companies (Cramer, 2017). There were other effects which were considered minor because their means were below the grand mean. The minor effects included poor ESPs resulting in conflict between the organisations and their communities (M = 3.2836; S = 1.1399) (Table 3). Other minor effects are that ESPs reduce cost in the long run but increases cost in the short run (M = 3.3758, S = .7841) and best ESPs may lead to positive public relations (Akuoko and Bour, 2013). It is also observed from the table that the effect of ESPs on the reputation people have for the organisation (M = 2.8221; S = 1.2058) is insignificant because it is far below the grand mean of 3.4830. Hence, whether or not these MFs develop and keep best ESPs, their reputation is not at risk.

Further examination and analysis revealed that there was a negative correlation (-0.6) between the cost of maintaining best of ESPs and the organisation's plans for improving them. The correlation coefficient was significant at 0.01 level. The negative correlation implies that as the cost of maintaining ESPs increases the MFs plans to improve, these ESPs decline (Table 6). This evidence thus corroborates the earlier findings by Maas et al. (2016) who discovered that ESPs decrease corporate's profit margins. As result of this correlation one can conclude that organisations are more likely to pay less attention to best ESPs since their improvement may have a negative effect on their financial base. This finding, according to Schaltegger et al. (2017), among others explains why many of MCs have successively failed environmental sustainability tests.

**Table 6:** The correlation between cost of Maintaining ESPs and the Plans for Improving ESPs.

		Cost of maintaining ESPs in the organisation	The organisation has plans for improving its E.S. Practices
Cost of maintaining ESPs in the organisation	Pearson Correlation	1	--0.550∗∗
	Sig. (2-tailed)		.000
	N	596	596
The organisation has plans for improving its E.S. Practices	Pearson Correlation	-0.550∗∗	1
	Sig. (2-tailed)	.000	
	N	596	596

Source: Authors' construct

∗∗ Correlation is significant at the 0.01 level (2-tailed).

## 4 Conclusions

The study has provided an insight into the nature of environmental sustainability practices of manufacturing organisations in the Ashanti region of Ghana. The data for the analysis was obtained from a sample of 600 respondents from 6 organisations which were listed by EPA for environmental sustainability assessment. The research intended to ascertain the nature, challenges, effects and the management of ESPs by manufacturing companies. The motivation for this study stemmed from the appalling outcome of the assessment of ESPs of MCs in the Ashanti by the EPA in 2009 and 2012. Therefore, the study sought to find out why almost all the manufacturing organisations in the region performed badly in that assessment. The premises by which the conclusion of this study is drawn are as follows: The research discovered that ESPs were developed by management for incorporation and renewal of incorporation, EPA inspections were considered as mere formality, most of the MCs do not have environmental protection department let alone having officers to man them; generally there is negative correlation between best ESPs and profit margin of MCs, so MCs engage in ESPs that are likely to have little or no effect on their profit and management of these practices is also found to be unreliable and ineffective. It is therefore, concluded that ESPs are considered as mere requirement for incorporation while EPA's usual inspections are seen as mere formalities and have no effect whatsoever on the growth and operations of the MCs. As a result, these MCs are not willing to commit resources toward environmental sustainability because they consider them as mere formality. This is said to have accounted for why most of the MCs in the Ashanti region have consecutively performed below standard on environmental sustainability test. The adoption of green technologies are recommended by MC's since they produce little wastes and promote more healthier lifestyles and wellbeing with low costs to companies.

## Declarations

### Author contribution statement

Kwame B. Bour: Conceived and designed the experiments; Performed the experiments; Analyzed and interpreted the data; Contributed reagents, materials, analysis tools or data; Wrote the paper.

Jones Asafo Akowuah: Performed the experiments; Analyzed and interpreted the data; Contributed reagents, materials, analysis tools or data; Wrote the paper.

Bernard Owusu Kwarteng: Performed the experiments; Analyzed and interpreted the data; Contributed reagents, materials, analysis tools or data.

### Funding statement

This research did not receive any specific grant from funding agencies in the public, commercial, or not-for-profit sectors.

### Competing interest statement

The authors declare no conflict of interest.

### Additional information

No additional information is available for this paper.
